# Pediatric Thyroid Cancer in Europe: An Overdiagnosed Condition? A Literature Review

**DOI:** 10.3390/diagnostics10020112

**Published:** 2020-02-19

**Authors:** Andreea-Ioana Stefan, Andra Piciu, Alexandru Mester, Dragos Apostu, Marius Badan, Claudiu-Iulian Badulescu

**Affiliations:** 1 2nd Pediatric Department Iuliu Hatieganu University of Medicine and Pharmacy, 400012 Cluj-Napoca, Romania; andreea.stefan24@gmail.com; 2 Department of Medical Oncology Iuliu Hatieganu University of Medicine and Pharmacy, 400012 Cluj-Napoca, Romania; 3 Department of Oral Health Iuliu Hatieganu University of Medicine and Pharmacy, 400012 Cluj-Napoca, Romania; alexandrumester@yahoo.com; 4 Department of Orthopedic Iuliu Hatieganu University of Medicine and Pharmacy, 400012 Cluj-Napoca, Romania; apostudragos@yahoo.com; 5 Department of Anatomy and Pathology Iuliu Hatieganu University of Medicine and Pharmacy, 400012 Cluj-Napoca, Romania; badan_marius@yahoo.com (M.B.); claubad05@yahoo.com (C.-I.B.)

**Keywords:** pediatric thyroid cancer, Europe, Chernobyl

## Abstract

Thyroid neoplastic pathology is the most common form of cancer associated with radiation exposure. The most common histopathological type of thyroid carcinoma is the differentiated thyroid cancer (these include papillary and follicular type), which represents over 90% of all cases, especially affecting girls rather than boys. Although patients are diagnosed in advanced stages as compared to adults, the prognosis of the disease is very good, with a 30-year survival rate of over 95% but post-therapeutic morbidity remains quite high. The treatment is based in particular on the therapeutic guidelines for adults, but as children have some histopathological and genetic characteristics of thyroid cancer, as well as different initial clinical presentations, we decided to review the literature on this pathology among the pediatric population, focusing on cases in Europe. The major interest is the impact of the Chernobyl accident.

## 1. Introduction

Pediatric thyroid cancer is one of the rarest endocrine tumors, yet the incidence rate increases by 1.1% per year. This increase is real, mostly because of the different environmental factors (ionizing radiation, volcanic activity, nitrate concentration in drinking water), unlike in the case of adults, where ultrasound screening plays an important role [1,2,3,4,5].

Thyroid neoplastic pathology is the most common form of cancer associated with radiation exposure. Studies of the population affected by the atomic bombs in Japan (Hiroshima and Nagasaki), the Marshall Islands, or those exposed to radiation released after the Chernobyl accident have demonstrated a close causal relationship between the occurrence of thyroid cancer and radiation exposure [6]. Thyroid cancer may also occur spontaneously, without any pre-existing thyroid pathology, or it may complicate a previous thyroid disease [7].

Since thyroid cancer is a rare disease in children, the diagnosis can be quite difficult initially. A high suspicion of malignancy arises in the presence of cervical adenopathy that appears in a non-infectious context, a palpable thyroid nodule or an asymmetric thyroid gland. An important step to support the diagnosis is imaging assessments, especially the thyroid ultrasound, which can confirm or deny the suspicion of the diagnosis by subsequently requiring fine needle aspiration (FNA) or surgery for histopathological evaluation (recommendation rating: B) [8].

The most common histopathological type of thyroid carcinoma is differentiated thyroid cancer (including papillary and follicular type), which represents over 90% of all cases, affecting more girls than boys [8,9,10]. Although pediatric patients are diagnosed in more advanced stages compared to adults, the prognosis of the disease is very good, with a 30-year survival rate of over 95% [1,10,11,12,13].

The treatment is based in particular on the therapeutic guidelines for adults. In 2015, the ATA (American Thyroid Association) published a guide for recommendations for the treatment of pediatric thyroid cancer [8]. Although the prognosis of pediatric thyroid cancer is excellent, post-therapeutic morbidity remains quite high, especially because of the occurrence of hypoparathyroidism, secondary to thyroidectomy, and recurrent laryngeal nerve damage. The iodine 131 treatment effect on the salivary, lacrimal, or gonadal glands can also be added as one of the complications [1,11,14].

Pediatric patients with thyroid cancer are treated according to the adult guidelines, and thyroid cancer is not classified differently in children compared to young adults [15,16,17,18]. Child thyroid cancer, however, does have some histopathological and genetic characteristics, as well as different initial clinical presentations. For example, children less than 10 years of age present more aggressive forms of the disease [19]. Therefore, we decided to review the literature on pediatric thyroid cancer, focusing on the European population, with the major interest being the impact of the Chernobyl accident [20,21].

The role of this study is to improve the understanding of pediatricians and oncologists of this pathology.

## 2. Materials and Methods

**Figure 1:**
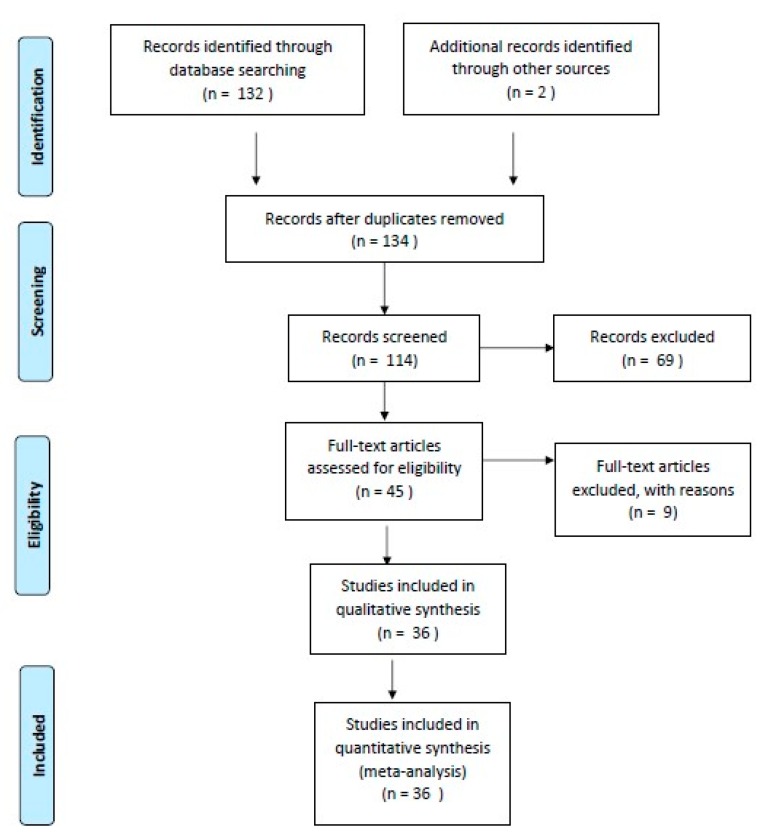
Selection criteria (PRISMA 2009 Flow Diagram).

We performed an electronic search on the PubMed database using the following terms: “pediatric”, “thyroid”, “cancer”, and “Europe”. In total, 132 articles (clinical trial and review) were obtained. After applying the filters “full text”, “humans”, “English”, and “French”, we had a selection of 114 articles. Articles that did not mention any history of irradiation for other conditions, a history of other malignancy, population migration (which can falsely increase the local incidence of thyroid cancer), and treatment of thyroid cancer (it will be part of a subsequent study) were excluded. Additionally, we performed a hand search including two studies. Finally, 36 articles were published between 1992 and 2018 (Figure 1). 

## 3. Results

The publications analyzed in this study are listed in Table 1.

Because the presence of medullary thyroid cancer in multiple endocrine neoplasia type 2 (MEN2) syndrome might involve screening of families, in order to evaluate the presence of thyroid nodules, proto-oncogene *RET* mutations, and serologic calcitonin values, we considered that the respective epidemiology was in fact influenced by direct medical intervention.

Most of these studies (72%) were retrospective, with only 5% prospective, and 17% were reviews; only 6% were case reports.

**Figure 2:**
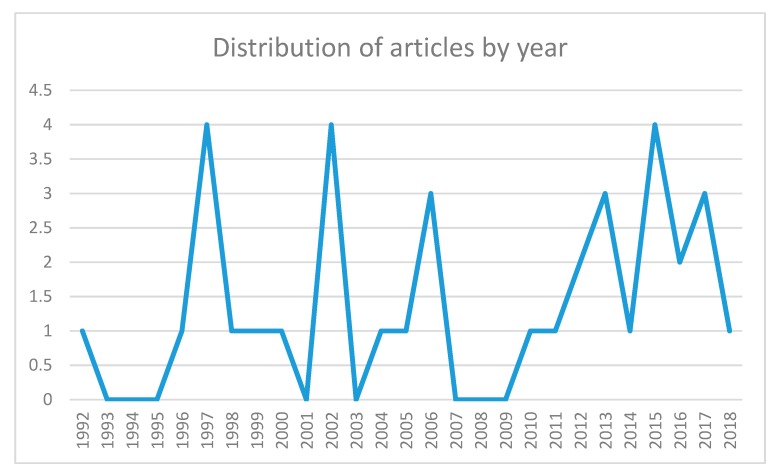
Graphical distribution of articles over time.

**Figure 3:**
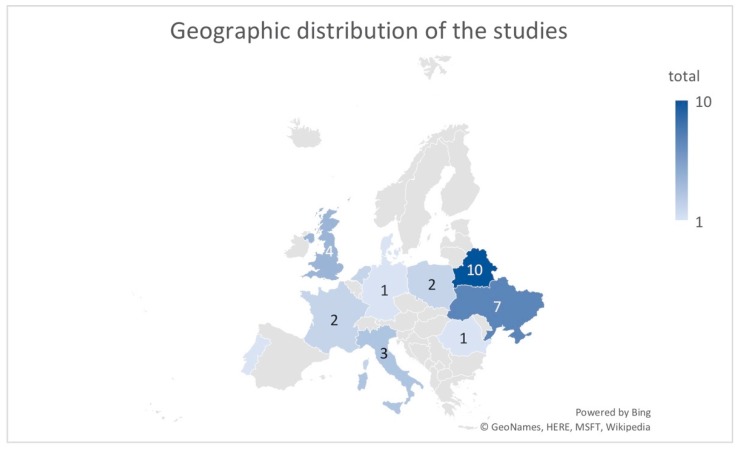
Geographic distribution of the studies in Europe.

Thyroid cancer in children is still a topic of interest, as shown in Figure 2. This topic has been intensively studied in Belarus and Ukraine, which are countries directly affected by the Chernobyl accident (Figure 3). Figure 2 demonstrates an unusual interest of scientists on this topic; the “saw” pattern of the distribution is related to 5-10-15 years after Chernobyl. Between these periods, studies are almost completely missing.

### 3.1. Epidemiology

After the April 1986 nuclear accident, pediatric thyroid cancer presented an increased incidence, especially in countries adjacent to Chernobyl. This increase in incidence is not determined by a more intensive initial screening but the emergence of more new cases, most likely determined by external factors (nuclear radiation) [22,24]. The new cases were found to be more aggressive, with significant local invasion and distant metastases [5,22,38]. The first studies did not conclude a causal relationship between irradiation and the onset of thyroid cancer. Subsequent studies have confirmed that it took at least 5 years from the time of irradiation and the onset of the disease. 

The irradiation path is important, whether it is external or by ingestion [26]. As the cause of thyroid cancer develops more and more because of radioisotope ingestion and less to external irradiation, this ingestion occurs in particular through the contamination of food (grass consumed by animals; subsequently, milk and dairy products in children’s nutrition). As the radioisotope accumulates, especially in the thyroid gland and not in other tissues, it justifies the thyroid cancer incidence increase and not of other malignancies [26,27,34,35]. Children were more susceptible to developing thyroid cancer due to increased thyroid function, increased milk intake from nutrition, as well as an increased minute-respiratory volume given by the high respiratory frequency in children compared to adults [24,34]. Also, it is important to underline that, compared with the adults, the percent of metastatic cases mainly in the lymph nodes at the initial presentation is higher, between 30% and 80% vs. 20% and 50% of adults and with pulmonary metastases in 9%–30% vs. 2%–9% of adults [42,43]. Additionally, there is better *NIS* expression and better response to radioiodine therapy (RAI) [44]. 

Chernobyl irradiation sources were ingestion of radioactive iodine with short half-life (I^131^), external exposure to radionuclides presented in soil (Ze^95^ + Nb^95^, Ru^103^, Ru^106^, Te^132^, I^132^, Ba^140^ + La^140^, Ce^141^ + Ce^144^), and ingestion of radioactive cesium (especially Cs^134^ and Cs^137^) [35].

Studies conducted in Japan among the survivors of the atomic bomb blasts or among the individuals who were irradiated at the thymus and face due to medical reasons found that radiation-induced thyroid neoplasms are closely related to sex (they encountered a higher incidence in women) and are inversely proportional to the age of exposure. These variations can be explained in part because younger people have a lower metabolic rate than older people and their tissue absorbs a higher number of radionuclides [31].

In countries affected by the nuclear explosion (Ukraine, Belarus), new cases of thyroid cancer were diagnosed at 4 to 10 years from the time of the Chernobyl accident, with an average of 6 years. It is known from other studies that a period of 5 years is needed between the nuclear accident and the occurrence of thyroid cancer in order to establish a causal relationship between the two events [24].

The incidence of thyroid cancer in children has markedly increased during the last 30 years in the areas directly affected by the Chernobyl accident; on the other hand, countries with no exposure to the nuclear clouds had similar trends of the incidence over the years. The annual incidence of thyroid cancer cases varies between 0.19/million children in England and Wales, 0.4 in Belarus before the Chernobyl accident, 1 in Norway, and 1.8 in the USA [2].

Denmark accounts for the largest proportion of thyroid cancer in children aged 10–14 and no changes in frequency have been observed between 1978 and 2014 [41].

The same trend is also found in the Netherlands, where there was an incidence of 0.4 cases of differentiated thyroid cancer/100.000 for the 0–4 years group and 1.5/100.000 in the 15–19 years group, between 1970–2013 [14].

A retrospective study conducted in 1991–2010 in Scotland highlights the emergence of 3.4 newly diagnosed cases/thyroid cancer in children [16].

The incidence of thyroid cancer in children is described in the literature as 0.5–1.2/million for children under 14 years of age and 4.4–11/million for children between 15 and 19 years of age, with growth in both Europe and America. In Portugal, it could not be described in the trend, specifically in a study carried out on 93 cases from 1964–2006 [19].

The incidence in Germany is described as 1/1 million (0–15 years) [9].

Italy, although not directly affected by the Chernobyl accident, describes an increasing incidence of thyroid cancer in children that is variable between the different regions of the countries (the highest incidence is found in the center: 12 cases/1 million; the lowest in the south: 9.1 cases/1 million). The biggest difference between the regions is seen especially in the age group 0–14 years, where the incidence in the center of the country (15.6 cases/1 million) is 4 times higher than in the south of Italy (3.7 cases/1 million). For the group aged 15–19 years, there was a significant increase in cases between 1988–2008 among girls [10]. It was hypothesized that volcanic activity could influence the occurrence of thyroid cancer cases in children, so in the study conducted in Sicily, in the period 2002–2009, several new cases occurring in volcanic areas were registered (1.4/100.000 girls, 0.5/100.000 boys) compared to non-volcanic areas (0.6/100.000 girls, 0.1/100.000 boys) while the literature describes a general incidence of 0.8/100.000 girls and 0.2/100.000 boys [3].

In the countries directly affected by the Chernobyl accident, the trend is increasing. The number of newly diagnosed cases in the period immediately following 1986 increased exponentially [4,5,13,31,33,37]. Those who were children at the time of the accident became young adults in 2002, determining the increase of incidence in this population, while there was a decrease of the incidence of thyroid cancer in young children. Between 1986 and 2002, 5000 cases of thyroid cancer were diagnosed in the population that was under 15 years or adolescents at the time of the accident, in the three affected countries of Belarus and Ukraine and the four intensely affected regions of Russia [13,35]. 

Between 1986 and 1996, the highest increase in incidence was recorded in Belarus, from 0.5/1 up to 30/1 million/year, while in Ukraine, it increased up to 3.4/1 million/year. In some areas of Belarus, the incidence even increased to 90 cases/million/year [24,34]. The incidence increase was found to be directly proportional to the level of radioactive contamination and inversely proportional to the age of the child. Children aged 0–5 years at the time of the accident are 3 times more likely to develop thyroid cancer than those aged 6–11. The incidence returns to pre-accident values among children born 6 months after the accident [4,24,37]. 

These assertions are also supported by the retrospective study published in 1992, which highlights the occurrence in Belarus in 1986–1991 of 101 new cases in children under 15 years while in 1976–1985, only nine new cases in children under 15 years were described [26].

In Poland, the trend was increasing. In the period 1972–1995, there were 23 cases (12 cases in 1972–1991, 0 cases in 1992–1993, 11 cases in 1994–1995) diagnosed and in the period 1996–2000, 37 new cases in children under the age of 19 were reported, leading to an incidence of 0.68/100.000 [2].

In Romania, the incidence increased 10 years after the accident; then 25 years after the accident, it became stationary in the evolution. The cases registered in the period 2005–2010 are 6.5 times more than those registered in the period 1991–1995, which suggests that the incidence increase was not due to screening but a consequence of the irradiation of the population following the Chernobyl accident [5].

All studies report a higher frequency of occurrence in girls compared to boys. This ratio is observed especially during adolescence. Mortality is low, and survival is reported in all cases at over 96% [1,2,3,4,5,6,7,8,9,10,11,12,13,14,15,16,17,18,19,20,21,22,23,24,25,26,27,28,29,30,31,32,33,34,35,36,37,38,39].

### 3.2. Histology

The histological changes found were similar to those reported in the literature, with predominant cases of papillary thyroid cancer [5,11,22,23,24,31].

Different studies compared spontaneous and radiation-induced thyroid cancer cases in order to highlight the impact of nuclear radiation on the appearance of thyroid cancer. Most radiation-induced cases have occurred in children who were under 5 years of age at the time of exposure to radiation [22,23,24]. These tumors were much more aggressive than those that appeared spontaneously, with a short latency period between the time of exposure and their onset, and histologically they were almost exclusively papillary thyroid carcinomas (predominantly follicular variants but pure papillary forms are present), but also cases of follicular thyroid cancer, medullary thyroid cancer, and rare cases of anaplastic thyroid cancer [5,22,23,24,26,29,31,40,41]. Medullary thyroid cancer occurs especially in patients with MEN2B syndrome [8,16].

An Italian study describing cases of thyroid cancer in children with sporadic subtypes subdivided the cases of papillary thyroid cancer as follows: Solid/trabecular variant 29%, classic variant 19%, microcarcinomas 16%, diffuse sclerosing variant 14%, follicular variant 10%, encapsulated variant 5%, and tall cells 7% [11]. There are no other randomized European studies referring to the subject of microcarcinomas in children. As it is well known that in adults the percent of microcarcinomas is high and continuously increasing, the subject has not yet been studied in a pediatric population. The low number might be due mainly to the very limited number of unjustified thyroidectomies compared with adults, a fact that needs further studies in the near future.

### 3.3. Genetics

*Ret/PTC* rearrangements are known and documented in the literature to be the cause of papillary thyroid cancer. These rearrangements mainly involve the 5′ and 3′ regions of the ret gene encoding the tyrosine kinase domain. Over 10 such rearrangements have been described, but the most common ones are *Ret/PTC1* and *Ret/PTC3*, whose prevalence varies from one study to the other. These variations depend on age, genetic background, histological subtype, and environmental factors, especially external or internal irradiation [16,32,34,45]. *RET* mutations are also involved in the onset of MEN2 and familial thyroid cancer syndromes [16,45]

The number of studies regarding the genetic mutations in thyroid cancer is increasing, some of them being multicenteric and performed in large cohorts, but these refer to the adult population. The publication of Shen, X. et al. [46] underlined that the long-observed age-associated mortality risk in PTC is dependent on the *BRAF* status; age is a strong, continuous, and independent mortality risk factor in patients with *BRAF V600E* mutation but not in patients with wildtype *BRAF.* These results question the conventional general use of patient age as a high-risk factor in PTC and call for differentiation between patients with *BRAF V600E* and wildtype *BRAF* when applying age to risk stratification and management of papillary thyroid cancer.

In addition, Melo, M. et al. [47] comments that the results of the mentioned study are relevant and deserve a thoughtful analysis because the molecular landscape of PTC in older patients is known to be different from that in their younger counterparts, and this difference was not taken into account. Furthermore, PTC mortality rates in both cohorts (wildtype *BRAF* and *BRAF* V600E) are low, which raises the question of whether a *BRAF*-modulated age stratification per se will be valuable in clinical practice. This debate confirms the need to adjust the studies strictly to a pediatric population and to modulate the strategies accordingly. 

**Figure 4:**
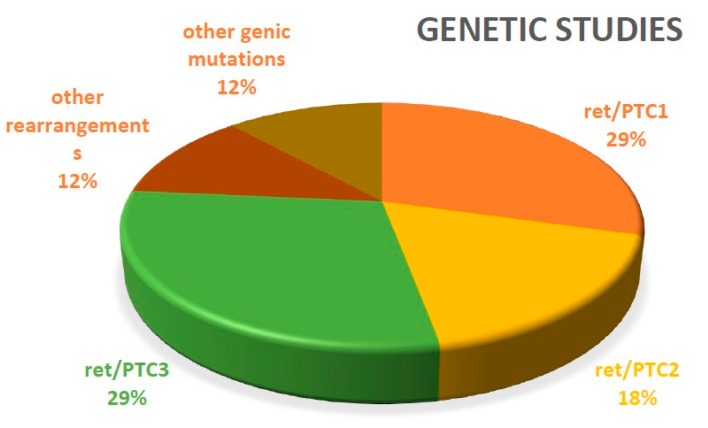
Genic rearrangements.

We can observe that 27.7% of the articles included in this review (10 articles from a total of 36) studied the genetics of thyroid cancer. The proportion of genic rearrangements is illustrated in Figure 4.

*Ret/PTC3* rearrangement has been found to occur primarily in cases of radiation-induced thyroid cancer, especially in papillary thyroid cancer (PTC), but may also occur in follicular ones. From the geographical point of view, these rearrangements are mainly found in patients in Belarus and Ukraine, as shown by comparative studies between this group of patients and patients in France, the USA, or Japan, where the frequency of the occurrence of these rearrangements was different [6,27,28,32,48]

In 2005, Cristofaro, J. et al. [32] carried out a comparative study between the people involved in cleaning the Chernobyl area after the accident, the people in Ukraine from areas not exposed to radiation, and those from the south of France not affected by radiation, all with thyroid pathology. The prevalence of *Ret/PTC* rearrangements in cases of papillary thyroid cancer differed across the three population groups as follows: 83.3% of Ukrainians involved in cleaning, 64.7% of unexposed Ukrainians, and 42.9% of unexposed French presented a *Ret/PTC1* predominance. The study was made on adults to be compared to the literature date referring to children, where *Ret/PTC3* rearrangements predominate. This study did not show a correlation between the ret rearrangements and the sex of the patient, the extent of the disease according to the TNM classification of malignant tumors (TNM), the age of diagnosis, or the age at which the patient was exposed to irradiation.

A study of patients from Ukraine, published in 2013 [37], analyzed the occurrence of other gene mutations than the Ret/PTC rearrangements already known in the literature. Nonoverlapping driver alterations were identified for each of the 26 cases exposed to radiation by the RNA-seq method. In total, 22/26 cases (84%) had fusion events, with at least 14 of them resulting from intrachromosomal rearrangements, further supporting the fact that spatial proximity favors the generation of recombination events after radiation exposure and DNA alteration. In total, 23/26 tumors express oncoproteins that activate mitogen-activated protein kinase (MAPK) signals, which confirms the critical role of activating this pathway in thyroid tumor formation, including the fusion of *ETV6-NTRK3* and *AGK-BRAF* kinases that encode activated kinases that can be targeted pharmacologically. Another study from Ukraine published in 2012 [49] consists of a report on a cohort of adult Ukrainian patients diagnosed with PTC from 2004 to 2008 following exposure at the age of 18 years or younger. The results suggest that this cohort has frequent *BRAF* mutation, *Ret/PTC1* rearrangement, and a low proliferation index. Furthermore, *BRAF* 1799T>A was underrepresented in PTCs with chronic lymphocytic thyroiditis (CLT), and cyclin A expression was associated with increased PTC tumor size.

In an article published in 2002 by Ito, the incidence of *Axl/Gas6* gene expression was exposed in the cases of papillary thyroid cancer in children diagnosed between 1993 and 1995 in Gomel, Belarus. It was shown that 76.5% of the cases had *Ax*l expression, in 70.6% of the cases had Gas expression, and the coexpression of *Axl/Gas6* was present in 100% of the cases as *Gas6* positive, and 84.6% in the cases *Axl* positive, while in the normal thyroid tissue, no expression of *Axl* or *Gas6* was detected. Strong expression of *Axl and Gas6* was observed in the tumor margins and in the vascular invasion while 8/10 cases (80%) with lymph node metastases had positive Axl in the primary tumor [30].

In 2010, Stein et al. published a study using samples collected from the Chernobyl tissue bank from patients operated on between 1999and 2002, studying the occurrence of copy number alteration in radiation-induced cancer. Thus, it describes a series of mutations identified in the tumor cells, pointing out that the genetic mechanism involved in the appearance of these diseases is much more complex than what is currently known. It recalled the role of *Ret/ELE1* fusion (an androgen receptor activator of *Ret/PTC3* transcription) as well as the importance of ESR*1* and ESR2 (estrogen receptor) expression in the proliferation of tumor thyroid cells (these being stimulated by an ESR1 agonist, reduced by ESR2 expression or by an ESR2 agonist) [34].

In a recent Italian study including 59 pediatric patients who underwent surgery for differentiated thyroid cancer (DTC) between 2000 and 2017, *Ret/PTC* rearrangements were analyzed and compared with adults.

*Ret/PTC* rearrangements were confirmed as the most frequent genetic alteration in childhood DTC (24.6%) and correlated with aggressive features. *BRAFV600E* was only identified in 16% of the pediatric DTCs while *NRASQ61R*, *NRASQ61K*, and *TERTC250T* mutations were very rare [50].

## 4. Discussions

Thyroid cancer among the pediatric population is part of the rare group of diseases, but it is important it is known by all health professionals, especially those who have direct contact with pediatric patients. The cases of thyroid cancer in children are increasing year by year, a trend observed throughout the world, especially in areas affected by radioactivity. This increase is real and is not attributed to intense imagistic screening [1,2,3,4,5].

So far, these cases have been managed according to the protocol for adults [51], but histopathological, genetic, and clinical differences have been observed between the different age groups in patients under 18; therefore, it is important to consider the development of specially designed treatment guides for children, even subdivided by age categories (child, adolescent).

Genetic studies that have highlighted the role of estrogen and androgen receptors in the proliferation of tumor cells have been performed, so that in the future, the development of hormonal therapy targeting these receptors might be considered. The role of hormones in inducing this condition can also be seen from the simple observation of the ratio of girls/boys, which is greater than 1 in adolescents and equal or slightly reversed in children under 10 years.

Stein et al. [34] studied the genetic mutations of patients with papillary thyroid cancer following exposure to radiation during the Chernobyl fallout tragedy and who were children at the time of the accident. According to the mentioned authors, gene expression analysis revealed that most of the altered genes were also perturbed in sporadic adult PTC; however, 141 gene expression changes were found to be unique to the post-Chernobyl tumors. The genes with the highest increases in expression that were novel to the pediatric post-Chernobyl tumors were *TESC, PDZRN4, TRAa/TRDa, GABBR2*, and *CA12.* The genes showing the largest expression decreases included *PAPSS2, PDLIM3, BEXI, ANK2, SORBS2*, and *PPARGCIA*; some of the mutations were unique to the radiation-induced profiles.

Genetic analyses revealed the occurrence of multiple punctiform mutations or gene rearrangements at the tumor level, indicating that this pathology is insufficiently known from the genetic point of view and further studies could help us to develop a gene therapy as an alternative to the current treatment. The role of the preoperative molecular testing of FNA was limited, mainly because the studies occurred during a long period of at least two decades, when there was clear unavailability of the method on a routine basis.

Fortunately, all studies report an excellent survival of over 96%, but secondary morbidity induced by the therapy is quite high (hypoparathyroidism, recurrent laryngeal nerve damage, pulmonary fibrosis). In this situation, the elaboration of alternative therapies or guidelines especially designed for children could help reduce the secondary morbidity to improve the quality of life. 

## Figures and Tables

**Table 1:** The articles included in this review.

	No.	First Author’s Name	Year of Publication	Country of Study	Study Type
**Screening**
	**1.**	Ito, M. [22]	1996	Belarus	Retrospective
	**2.**	Bucsky, P. [9]	1997	Germany	Review
	**3.**	Nikiforov, Y.E. [23]	1997	Belarus	Retrospective
	**4.**	Schwenn, M.R. [24]	1997	Europe	Review
	**5.**	Furmanchuk, A.W. [25]	1992	Chernobyl area	Retrospective
	**6.**	Zimmerman, D. [26]	1997	Europe	Review
	**7.**	Nikiforov, Y.E. [27]	1998	Belarus	Retrospective
	**8.**	Thomas, G.A. [6]	1999	Belarus and Ukraine	Prospective
	**9.**	Fenton, C.L. [28]	2000	USA, Washington	Retrospective
	**10.**	Marks, S. [29]	2002	Belarus, Ukraine, Canada	Case presentation
	**11.**	Ito, M. [30]	2002	Belarus	Retrospective
	**12.**	Niedziela, M. [7]	2002	Poland	Case presentation
	**13.**	Moysich, K. [31]	2002	Belarus, Ukraine	Review
	**14.**	Niedziela, M. [2]	2004	Poland	Prospective
	**15.**	Cristofaro, J. [32]	2005	Ukraine, France	Retrospective
	**16.**	Stiller, C.A. [33]	2006	UK, Europe	Retrospective
	**17.**	Collini, P. [11]	2006	Italy	Retrospective
	**18.**	Stein, L. [34]	2010	UK	Retrospective
	**19.**	Cardis, E. [35]	2006	Chernobyl area	Review
	**20.**	Tuttle, R. [17]	2011	Russia, Ukraine, Belarus	Review
	**21.**	Piciu, D. [5]	2012	Romania	Retrospective
	**22.**	Redlich, A. [36]	2012	Germany	Retrospective
	**23.**	Ricarte-Filho, J. [37]	2013	Ukraine	Retrospective
	**24.**	Pisani, P. [10]	2013	Italy	Retrospective
	**25.**	Reiners, C. [13]	2013	Ukraine	Retrospective
	**26.**	Fridman, M. [38]	2014	Belarus	Retrospective
	**27.**	Drozd, V. [4]	2015	Belarus	Retrospective
	**28.**	O Hara, C. [12]	2015	UK	Retrospective
	**29.**	Silva-Vieira, M. [19]	2015	Portugal	Retrospective
	**30.**	Douglas, C.M. [16]	2015	Scotland	Retrospective
	**31.**	Desandes, E. [39]	2016	France	Retrospective
	**32.**	Hesselink, M. [14]	2016	The Netherlands	Retrospective
	**33.**	Bogdanova, T. [40]	2017	Ukraine, Japan	Retrospective
	**34.**	Russo, M. [3]	2017	Sicily	Retrospective
	**35.**	Dekker, B. [1]	2018	Europe	Retrospective
	**36.**	Grønhøj, C. [41]	2018	Denmark	Retrospective
